# Effects of auricular point acupressure on inflammatory signaling in chronic low back pain: a secondary analysis of a randomized controlled trial

**DOI:** 10.1186/s12906-025-05227-9

**Published:** 2025-12-29

**Authors:** Nada Lukkahatai, Hongyu Wang, Xinran Huang, Wanqi Chen, Hulin Wu, Jennifer Kawi, Zhiyin Yu, Constance M Johnson, Paul Christo

**Affiliations:** 1 School of Nursing, Johns Hopkins University, 525 North Wolfe Street, Baltimore, MD 21205 USA; 2 McGovern Medical School, University of Texas Health Science Center at Houston, Houston, TX USA; 3 Cizik School of Nursing, University of Texas Health Science Center at Houston, Houston, TX USA; 4 School of Public Health, University of Texas Health Science Center at Houston, Houston, TX USA; 5 School of Medicine, Johns Hopkins University, Baltimore, MD USA

**Keywords:** Auricular point acupressure, Ear acupressure, Chronic pain, Inflammatory biomarkers, Low back pain

## Abstract

**Objective:**

Auricular point acupressure (APA) has shown promise for pain relief, but its biological mechanisms remain unclear. This study examined the effects of APA on inflammatory cytokines and explored the relationships between changes in inflammatory markers and clinical outcomes in older adults with chronic low back pain (cLBP).

**Method:**

This secondary analysis utilized plasma samples from a three-arm randomized controlled trial (RCT) (NCT03589703) with 272 participants aged 65 years and older with cLBP. The participants were randomly assigned (1:1:1) to the targeted APA (T-APA), nontargeted APA (NT-APA), or education control (CG) groups for four weeks. Cytokine levels were measured at baseline, post-intervention, and at the one- and three-month follow-ups using multiplex assays. RNA sequencing and quantitative PCR (qPCR) were performed to assess changes in inflammatory gene expression. The primary outcome was the change in inflammatory cytokine levels, and the secondary outcomes included self-reported pain and physical function.

**Results:**

A total of 272 participants were randomized (T-APA: 92, NT-APA: 91, CG: 89), with 43.4% completing follow-up. The participants in the T-APA group showed significant reductions in the levels of proinflammatory cytokines (IL-2, IL-8, and TNF-α) and increases in the levels of the anti-inflammatory cytokine TGF-β. These changes correlated with improvements in pain and physical function.Gene expression analysis suggested the modulation of *CXCL8* and *GPS2*, indicating the potential influence of APA on neuroimmune signaling. The NT-APA and CG groups did not show similar benefits. No serious adverse events were reported.

**Conclusion:**

These findings suggest that APA has immune-modulating effects on reducing inflammation and improving pain outcomes in older adults with cLBP. However, the discrepancies between the RNA sequencing and qPCR results warrant further investigation into the molecular mechanisms of APA. Future studies should incorporate longer follow-up periods and larger sample sizes to validate these findings.

**Trial registration:**

Trial ID: NCT03589703 (registration date: May 22, 2018).

**Supplementary Information:**

The online version contains supplementary material available at 10.1186/s12906-025-05227-9.

## Introduction

Chronic low back pain (cLBP) is a widespread health concern that particularly affects older adults and is one of the leading causes of disability worldwide. In the United States, cLBP disproportionately impacts individuals aged 65 and older, leading to mobility limitations, decreased quality of life, and increased reliance on healthcare services [1]. The economic burden of cLBP in the United States is substantial, with total costs estimated at approximately $40 billion, including direct healthcare expenditures and lost productivity [2]. While pharmacological treatments such as opioids and NSAIDs are widely used, they pose risks, including dependence, gastrointestinal complications, and cardiovascular issues [3]. Nonpharmacological approaches, such as physical therapy, cognitive‒behavioral therapy (CBT), and spinal manipulation, offer alternatives but present challenges. Exercise and physical therapy require consistent participation, which can be difficult due to mobility limitations and pain flares [4]. CBT helps modify pain perception but requires sustained engagement and access to trained providers [5]. Spinal manipulation and massage therapy provide temporary relief but may be costly and difficult to access [6]. Given these barriers, there is a need for noninvasive, accessible, and sustainable pain management strategies for older adults with cLBP.

Auricular point acupressure (APA) is a noninvasive intervention rooted in auricular microsystem theory, which posits that the external ear is a microsystem reflecting the entire body [7]. By applying pressure to specific auricular points, the APA is believed to activate neurological and physiological responses that may contribute to pain relief. Recent clinical trials have investigated the effects of auricular therapies, including APA and auricular acupuncture, on various pain conditions. Some studies have reported significant reductions in pain intensity and improvements in function, particularly in musculoskeletal disorders [8–11]. A meta-analysis including 13 studies of auricular acupressure and acupuncture for pain management reported that auricular acupressure and acupuncture provided significant pain reduction compared with NT-APA or control groups (standardized mean difference [SMD] of -1.59, 806 participants across 13 trials) [12]. Despite growing evidence supporting the clinical benefits of APAs, the understanding of their biological mechanisms, particularly in relation to the inflammatory processes that contribute to chronic pain, is limited.

Inflammation plays a crucial role in pain pathophysiology, with proinflammatory cytokines such as IL-2, IL-6, and IL-8 associated with heightened pain sensitivity and chronicity. Conversely, anti-inflammatory cytokines such as TGF-β may relieve pain by modulating immune responses [11, 13, 14]. The interplay among cytokines, neuropeptides, and pain pathways suggests that APA may exert its effects through immune regulation, reducing inflammation while improving pain-related outcomes. Preliminary studies on auricular therapies indicate a potential link between acupressure and cytokine modulation [11, 15, 16], although findings remain inconsistent owing to methodological variations, small sample sizes, and differences in intervention protocols. Further investigation is needed to establish a clearer understanding of the immune-modulating effects of APA in chronic pain patients.

To address gaps in understanding the biological mechanisms of APA effectiveness, we conducted a secondary analysis of an RCT evaluating the effects of targeted APA (T-APA) on inflammatory markers in older adults with cLBP. Given the role of inflammation in pain pathophysiology, this study examined changes in inflammatory cytokine levels across multiple time points to assess the potential immune-modulating effects of APA and their associations with self-reported pain intensity and physical function. This study builds on the parent trial [17], which demonstrated the clinical efficacy of APA and established minimal clinically important difference (MCID) thresholds for pain and function. The present analysis focuses on biological mechanisms, exploring whether changes in inflammatory cytokines correspond with the observed clinical improvements.

## Methods

This study is a secondary analysis of a three-arm, parallel-group, randomized controlled trial (NCT03589703; Registration date May 22, 2018) conducted to assess the effects of T-APA, NT-APA, and CG on inflammatory biomarkers and pain-related outcomes in older adults with cLBP.

### Study design and participants

The present study analyzed plasma collected from a 3-arm randomized controlled trial. The study participants were individuals aged 65 and older, had been diagnosed with cLBP for at least three months or self-reported intermittent pain for at least half of the day for six months or longer, had the worst pain intensity in the past seven days of at least 4 on a 0–10 numerical rating scale (NRS), were able to communicate in English, and had intact cognition (Mini-Mental State Examination > 24) [18]. Individuals with any of the following conditions were excluded: (1) diagnosed with malignant or autoimmune diseases (e.g., rheumatoid arthritis); (2) compression fractures with known causes (e.g., osteoporosis, spondylolysis, or spondylolisthesis); (3) pain in other parts of the body that is more severe than cLBP; (4) use of hearing aids; and (4) self-reported allergic reactions to adhesive tape (used to secure seeds to ear points).

The study protocols were approved by the Institutional Review Board of the Johns Hopkins School of Medicine (IRB00175409), and protocol details were registered and published [19]. All participants provided written informed consent. The study recruitment period ranged from June 26, 2018, to March 29, 2023. This cytokine analysis represents a secondary analysis using data from the parent RCT [17]. Although MCID thresholds for pain (≥ 1.5-point reduction) and physical function (≥ 2.5-point improvement) were established in the parent study, they were not applied in this mechanistic analysis because the primary objective was to examine cytokine modulation and its associations with clinical outcomes rather than re-evaluate clinical response thresholds.

### Randomization and blinding

The participants were randomized using a computer-generated sequence (1:1:1 ratio), with allocation concealment via sequentially numbered, opaque, sealed envelopes. Due to the nature of the intervention, blinding was not feasible for the interventionists and participants, but the outcome assessors and laboratory technicians were blinded to the treatment allocation.

### Intervention

The intervention in this 3-arm randomized controlled trial consisted of four weeks of weekly 15-minute sessions of interventionist-administered APA by trained APA interventionists, followed by five days of self-administered stimulation on prescribed ear points at home: (a) standardized ear points specific for cLBP (T-APA); (b) standardized ear points nonspecific for back pain (NT-APA); or (c) education control (CG). The detailed intervention protocol has been published elsewhere [19].

### Outcome measures

The study outcomes were cytokine concentrations and gene expression levels in blood samples collected at baseline (T1), post-intervention (T2, 4 weeks), and at the 1-month (M1) and 3-month (M3) follow-ups after the intervention. The participants’ peripheral blood samples were collected in an ethylenediamine tetraacetic acid (EDTA)-coated tube and PAXgene blood tubes (PreAnalytix/Qiagen) via standard phlebotomy procedures at T1, T2, M1, and M3. The samples in the EDTA tubes were processed immediately for plasma collection through centrifugation. The plasma was stored at -80 °C until use. The RNA extraction was performed using PAXgene Blood RNA Kits (Qiagen), ensuring A260/A280 values between 1.8 and 2.2 and genomic DNA contamination ≤ 1.0% (w/w). The RNA concentration and purity were assessed using a NanoDrop™ one spectrophotometer (Thermo Fisher Scientific, Waltham, MA, USA).

#### Plasma inflammatory cytokines

Cytokine analysis was performed by a blinded technician at the Oncology Center Laboratory Core, Sidney Kimmel Comprehensive Cancer Center at Johns Hopkins University, using a Luminex multiplex assay for 13 cytokines (IL-1α, IL-1β, IL-2, IL-4, IL-6, IL-8, IL-10, IL-12, IL-13, IL-17, IFN-γ, and TNF-α) and a single plex assay for TGF-β. All samples were run in duplicate, and a five-parameter regression formula was used to calculate the sample concentrations based on the standard curves. These assays typically exhibit high precision and reproducibility (84.5% sensitivity, 98% specificity; 92% of the patients in the active disease group are correctly classified from a cross-validation serum set) [20].

#### RNA sequencing (RNA-seq) and data processing

To confirm the change in protein level results at the gene expression level, RNA samples from 9 participants in the T-APA (*n* = 6) and CG (*n* = 3) groups were selected on the basis of the availability of complete blood collection and cytokine analysis results at all three time points (T1, T2, and M1). The NT-APA group was excluded from RNA analysis and downstream target gene expression analysis to focus on biologically relevant changes associated with the specificity of the intervention effects.

The RNA samples were processed at the UTHealth Cancer Genomics Center, including library preparation, quality control (QC), and sequencing. RNA-sequencing (RNA-seq) data were generated using an Illumina NextSeq 550 in 75 bp paired-end mode. The raw mRNA sequence reads were pre-processed with TrimGalore to remove bases with quality scores below 20 and adapter sequences. Clean reads were aligned to the reference genome Human Genome Reference Consortium Build 38, Version 83 (GRCh38.83) using Hierarchical Indexing for Spliced Alignment of Transcripts 2 (HISAT2). Genes with counts less than the number of samples were removed for analysis. The package DESeq2 in R was used to analyze the differentially expressed genes. Paired comparisons were conducted within and between groups, with multiple testing corrections performed using the Benjamini–Hochberg method. Significantly differentially expressed genes were identified based on an absolute fold change ≥ 1.5 and an adjusted p value ≤ 0.1.

#### Differential gene expression analysis (real-time PCR)

To validate the RNA-seq results, we used quantitative PCR (qPCR) to measure the expression levels of the differentially expressed genes identified. A total of 40 participants from the T-APA (*n* = 19) and CG (*n* = 21) groups were included in the downstream analysis. For cDNA synthesis, 500 *n*g of total RNA was reverse transcribed using the iScript cDNA Synthesis Kit (Bio-Rad, Hercules, CA, USA) according to the manufacturer’s instructions. Quantitative polymerase chain reaction (Q-PCR) was conducted on a Bio-Rad CFX Duet Real-Time PCR System with CFX Maestro software. The reactions were set up using the SsoAdvanced Universal Probes Supermix (Bio-Rad). Specific PrimePCR™ probes for *CXCL8*, *GPS2*, and *RSAD2* and the housekeeping gene *ACTB* were purchased from Bio-Rad. The thermal cycling conditions included initial denaturation at 95 °C for 2 min, followed by 40 cycles of 95 °C for 5 s and 60 °C for 30 s. Relative expression (fold changes) levels of *CXCL8*,* GPS2*, and *RSAD2* were normalized to those of *ACTB* using the normalized expression (ΔΔCq) method across different time points for both the APA and control groups. All samples were run in triplicate, and no template controls were included to verify the absence of contamination. The amplification efficiencies for all the genes exceeded 99.5%. Data analysis was performed using CFX Maestro software, and statistical significance was assessed using appropriate statistical methods.

#### Self-reported Pain and Physical Function

Pain and physical function measures followed the minimum dataset recommended by the National Institutes of Health (NIH) Pain Consortium’s Task Force on Research Standards for cLBP [21]. Pain severity was assessed using a numerical rating scale (NRS) ranging from 0 (no pain) to 10 (worst pain imaginable) based on the worst pain rating in the past seven days. Physical function was measured using the Roland‒Morris Disability Questionnaire (RMDQ), a 24-item questionnaire assessing functional limitations associated with cLBP, with higher scores indicating lower function and greater disability [22].

### Sample size and power analysis

Power analysis was conducted using the smaller effect size observed for function (Cohen’s d = 0.65) than for pain intensity (Cohen’s d = 1.28) at the one-month follow-up [9]. With a sample size of 272 participants, the study was designed to have a power of 0.90 to detect significant differences among the three groups, considering an attrition rate of 30%.

### Statistical analysis

The effects of APA on inflammatory cytokine concentrations were analyzed over time at baseline, post-intervention, and at the one- and three-month follow-ups. Each biomarker was examined individually and within two predefined latent variables representing proinflammatory and anti-inflammatory cytokines. Normality assumptions were assessed, and when the data did not meet the normality criteria, the Kruskal‒Wallis test was applied for multiple comparisons across groups. Because cytokine values exhibited severe censoring (near or above 50%) due to values below the detection limit, the generalized Wilcoxon score test was used to evaluate the effects of APA on inflammatory cytokine concentrations. This nonparametric method was used to handle highly censored data and ensure robustness of inference [23]. To account for repeated measures and individual variability, changes in biomarker levels were also analyzed using a generalized linear model (GLM), where values were categorized as increases or decreases from baseline. Cytokine data were summarized at each time point using the Kaplan‒Meier method, with the results reported as medians and interquartile ranges (IQRs).

Because of the substantial proportion of missing cytokine data across later time points, mixed-model analysis of variance could not be robustly applied. Instead, a nonparametric two-step approach was used. Within-group changes over time were first examined using the paired Prentice‒Wilcoxon test to identify cytokines showing significant temporary change within each intervention. For those cytokines,, between-group differences, which were subsequently evaluated using the generalized Wilcoxon score test. This strategy was selected to accommodate non-normal distributions and uneven data availability while maintaining the interpretability of longitudinal trends.

To evaluate the relationships between changes in clinical outcomes (pain and physical function) and inflammatory markers, Spearman’s rank correlation coefficient was first used to assess associations. For cytokines with significant within-group differences, one-way analysis of variance (ANOVA) was conducted as an exploratory analysis to compare mean cytokine changes across groups. The number and percentage of participants who responded to cytokine changes were summarized at each time point within each group. Due to the exploratory nature, the study aimed to identify preliminary immune-modulating patterns; p-values were not adjusted for multiple comparisons. Applying such corrections in small-sample, cytokine-based analyses can inflate Type II error and obscure potential biological signals. This approach was intended to balance sensitivity and interpretability in hypothesis-generating analyses, and results should therefore be viewed as preliminary.

### Missing data and sensitivity analysis

Given the high rate of missing blood samples, the primary analysis focused on within-group comparisons and was considered exploratory. Sensitivity analyses were conducted by imputing missing values based on detection limits. Values lower than the detection threshold were substituted with one-half of the lower limit, a method that, while potentially introducing bias [24], allowed the use of familiar summary statistics such as means and 95% confidence intervals.Paired t-tests were performed to validate the consistency of findings across imputed and observed datasets.

### Safety monitoring

Adverse events, including skin irritation, redness, and swelling, were monitored throughout the study. No serious adverse events were reported, and minor discomfort was observed in 1.1% of the participants, resolving spontaneously.

## Results

**Fig. 1:**
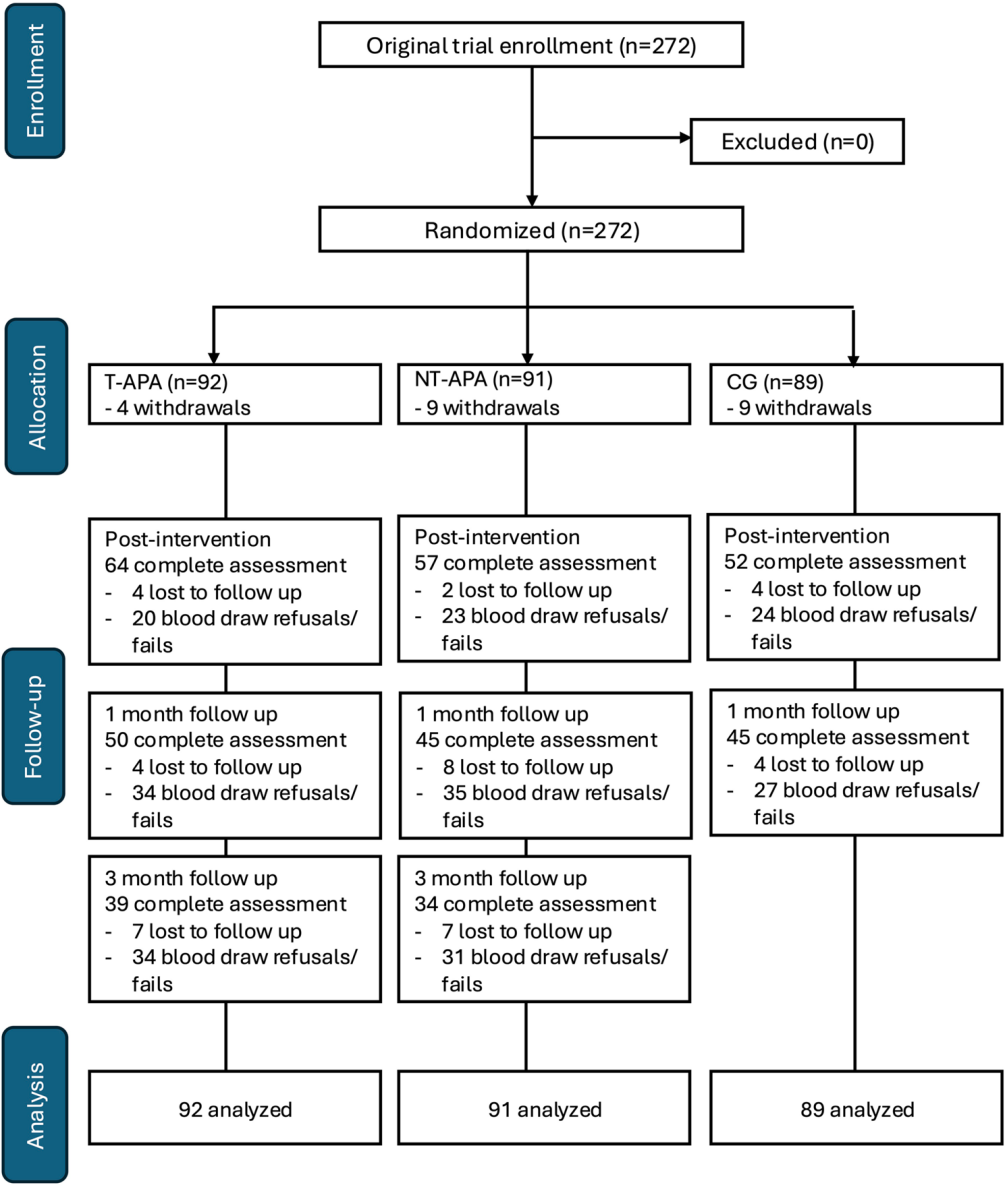
Modified CONSORT diagram for the secondary analysis focusing on inflammatory biomarkers and clinical outcomes. The full participant flow for the primary study is detailed in [17]

In the primary trial, 700 individuals were screened, with 178 found ineligible, 122 unable to be reached, and 128 declining to participate, resulting in 272 participants being enrolled and randomized into one of three groups: T-APA (*n* = 92), NT-APA (*n* = 91), and CG (*n* = 89). The full participant flow for the trial, including recruitment, allocation, and follow-up rates, has been reported in the primary outcomes paper. This secondary analysis focuses on a subset of participants with available biomarker data and examines the effects of APA on inflammatory cytokines in older adults with cLBP. A modified CONSORT diagram (Fig. 1) is provided to illustrate participant flow specific to this analysis, highlighting exclusions due to missing cytokine and gene expression data.

A total of 272 participants were enrolled in the study, with 92 in the T-APA group, 91 in the NT-APA group, and 89 in the CG group. The mean age of the participants was 70.0 years (SD = 7.0), with a similar distribution across groups. The majority of participants were female (64.0%), whereas 35.3% were male, and 0.7% did not specify their gender. The racial composition included 60.3% Black or African-American, 36.7% White, and 4.4% other or multiple races. Most participants (80.1%) were not Hispanic or Latino, 1.5% identified as Hispanic or Latino, and 18.4% did not report their ethnicity. The mean BMI was 31.0 (SD = 7.7), indicating that the obese population was overweight. With respect to education, 36.8% had a high school education or less, 17.6% had some college education, and 45.6% held a college degree or higher. In terms of employment status, 86.0% were unemployed or retired, whereas 12.1% were employed. Opioid use was reported by 44.5% of the participants, with the highest proportion in the NT-APA group (49.5%). At baseline, the mean pain intensity score on the NRS (range 0–10) was 7.2 (SD = 1.7), indicating moderate to severe pain, whereas the mean RMDQ (range 0–24) score was 12.3 (SD = 5.8), reflecting moderate functional impairment (Table 1).

**Table 1:** Modified demographics and baseline characteristics (*N* = 272). The participants’ demographics and baseline characteristics are detailed in [17]

Characteristics		T-APA (*n* = 92)	NT-APA (*n* = 91)	Control (*n* = 89)	Overall (*n* = 272)
Age (years), Mean (SD)		70.2 (6.5)	68.7 (7.1)	71.1 (7.2)	70.0 (7.0)
Gender, n (%)	Female	59 (64.1%)	56 (61.5%)	59 (66.3%)	174 (64.0%)
	Male	32 (34.8%)	35 (38.5%)	29 (32.6%)	96 (35.3%)
	Unspecified	1 (1.1%)	0 (0)	1 (1.1%)	2 (0.7%)
Race, n (%)	Black or African-American	51 (55.4%)	58 (63.7%)	55 (61.8%)	164 (60.3%)
	White	39 (42.4%)	28 (30.8%)	30 (33.7%)	97 (36.7%)
	Others/Multiple Races/unreport	2 (2.2%)	6 (6.6%)	4 (4.5%)	12 (4.4%)
Ethnicity, n (%)	Not Hispanic or Latino	84 (91.3%)	67 (73.6%)	67 (75.3%)	218 (80.1%)
	Hispanic or Latino	1 (1.1%)	1 (1.1%)	2 (2.2%)	4 (1.5%)
	Unknown or Not Reported	7 (7.6%)	23 (25.3%)	20 (22.5%)	50 (18.4%)
BMI, Mean (SD)		31.3 (8.5)	31.1 (7.7)	30.6 (7.0)	31.0 (7.7)
Education, n (%)	High school or less	27 (29.3%)	36 (39.6%)	37 (41.6%)	100 (36.8%)
	Some college	20 (21.7%)	17 (18.7%)	11 (12.4%)	48 (17.6%)
	College or higher	45 (48.9%)	38 (41.8%)	41 (46.1%)	124 (45.6%)
Employment, n (%)	Employed	9 (9.8%)	11 (12.1%)	13 (14.6%)	33 (12.1%)
	Unemployed/retired	80 (87.0%)	79 (86.8%)	75 (84.6%)	234 (86.0%)
	Unknown	3 (3.3%)	1 (1.1%)	1 (1.1%)	5 (1.8%)
Opioid Use, n (%)	Yes	44 (47.8%)	45 (49.5%)	32 (36.0%)	121 (44.5%)
	No/not sure	48 (52.3%)	46 (50.5%)	57 (64.0%)	151 (55.5%)
Baseline condition, Mean (SD)	Pain intensity (NRS)	7.4 (1.7)	7.0 (1.8)	7.3 (1.7)	7.2 (1.7)
	Physical Function (RMDQ)	12.4 (5.3)	12.6 (6.2)	12.0 (5.9)	12.3 (5.8)

*T-APA* Targeted-APA, *NT-APA* Non-targeted APA, *SD* Standard, *NRS* Numerical rating scale (score range from 0 no pain to 10 worse pain possible), *RMDQ* Roland‒Morris Disability Questionnaire (score range 0 no disability to 24 severe disability)

### Effect of APA on plasma cytokine levels over time

Using the paired prentice‒Wilcoxon test, we assessed within-group changes in cytokine levels from baseline across different time points (T2, M1, and M3). The T-APA group demonstrated a significant reduction in the levels of multiple proinflammatory cytokines (IFN-γ, IL-12p40, IL-8, and IL-2; all *p* < 0.05) at T2 and a significant increase in the level of anti-inflammatory cytokine TGF-β (*p* = 0.04) (Table 2), showing a greater anti-inflammatory effect compared with the NT-APA and control groups. These changes suggest an immediate anti-inflammatory response of the APA when stimulating the ear points targeted for the low back pain condition. However, these effects were not sustained beyond one month post-intervention (*p* > 0.05 at M1 and M3). In contrast, the NT-APA group exhibited a mixed cytokine response, with significant increases in proinflammatory cytokines (IL-6 at T2: *p* = 0.01, M1: *p* = 0.05; IL-12 (p70) at M3: *p* = 0.03) but also increases in anti-inflammatory markers (TGF-β at T2: *p* = 0.04, IL-4 at M3: *p* = 0.01) (Table 2). These findings suggest that stimulation at ear points that do not targeting the low back area may modulate immune responses. However, the pattern of cytokine changes was inconsistent across time points, and the intervention did not demonstrate a clear anti-inflammatory effect comparable to that of T-APA. The CG group exhibited no significant improvements in the levels of inflammatory markers, with the level of IL-12 (p70) increasing at M3 (*p* = 0.03), indicating worsening inflammation over time (Table 2).

**Table 2:** Change from baseline in each cytokine by group and time, mean (95% CI) ( *p *value)

Cytokine	T-APA			NT-APA			CG	
	T2 (*n* = 64)	M1 (*n* = 50)	M3 (*n* = 39)	T2 (*n* = 57)	M1 (*n* = 45)	M3- (*n* = 34)	T2 (*n* = 52)	M1 (*n* = 45)
Pro-inflammatory								
IFNg	**-0.15 (-0.28**,** -0.02) (0.02)**	-0.03 (-0.32, 0.26) (0.86)	-0.07 (-0.50, 0.35) (0.72)	0.20 (-0.01, 0.41) (0.07)	0.21 (-0.09, 0.51) (0.16)	0.28 (-0.04, 0.59) (0.08)	0.20 (-0.07, 0.46) (0.14)	0.06 (-0.24, 0.36) (0.70)
IL-12(p40)	**-0.11 (-0.22**,** -0.00) (0.05)**	-0.03 (-0.19, 0.13) (0.74)	-0.12 (-0.37, 0.12) (0.32)	-0.06 (-0.28, 0.16) (0.57)	0.08 (-0.16, 0.32) (0.51)	-0.16 (-0.52, 0.20) (0.38)	0.06 (-0.16, 0.27) (0.60)	0.07 (-0.16, 0.29) (0.56)
IL-12(p70)	-0.04 (-0.11, 0.03) (0.24)	0.03 (-0.14, 0.19) (0.75)	0.14 (-0.18, 0.46) (0.38)	0.14 (-0.10, 0.38) (0.24)	0.20 (-0.07, 0.47) (0.15)	**0.12 (0.01**,** 0.23) (0.03)**	0.12 (-0.12, 0.35) (0.32)	0.09 (-0.24, 0.42) (0.58)
IL-17 A	-0.03 (-0.10, 0.03) (0.31)	0.01 (-0.04, 0.05) (0.69)	0.11 (-0.09, 0.32) (0.26)	-0.02 (-0.15, 0.11) (0.75)	0.00 (-0.09, 0.10) (0.96)	-0.05 (-0.22, 0.11) (0.50)	0.03 (-0.03, 0.09) (0.35)	0.06 (-0.02, 0.14) (0.16)
IL-1a	-0.04 (-0.14, 0.06) (0.41)	0.06 (-0.05, 0.18) (0.25)	0.09 (-0.10, 0.29) (0.34)	0.02 (-0.15, 0.19) (0.82)	0.07 (-0.08, 0.22) (0.37)	-0.07 (-0.29, 0.16) (0.56)	0.06 (-0.06, 0.18) (0.34)	0.03 (-0.10, 0.15) (0.67)
IL-1b	-0.06 (-0.16, 0.04) (0.21)	-0.03 (-0.18, 0.12) (0.67)	-0.02 (-0.24, 0.20) (0.87)	0.06 (-0.18, 0.30) (0.61)	0.14 (-0.06, 0.34) (0.16)	-0.06 (-0.26, 0.13) (0.53)	0.08 (-0.07, 0.24) (0.28)	0.08 (-0.15, 0.30) (0.49)
IL-6	-0.07 (-0.26, 0.12) (0.48)	0.15 (-0.07, 0.38) (0.18)	-0.18 (-0.42, 0.06) (0.14)	**0.35 (0.10**,** 0.61) (0.01)**	**0.29 (0.01**,** 0.56) (0.05)**	0.33 (-0.05, 0.71) (0.08)	0.03 (-0.16, 0.23) (0.75)	0.16 (-0.19, 0.52) (0.36)
IL-8	**-0.15 (-0.29**,** -0.01) (0.04)**	-0.10 (-0.29, 0.09) (0.29)	-0.13 (-0.36, 0.10) (0.27)	0.09 (-0.07, 0.25) (0.26)	0.11 (-0.13, 0.35) (0.37)	0.21 (-0.08, 0.51) (0.15)	0.12 (-0.01, 0.25) (0.07)	0.02 (-0.23, 0.26) (0.90)
TNFa	-0.05 (-0.11, 0.00) (0.06)	-0.02 (-0.14, 0.11) (0.81)	-0.06 (-0.27, 0.15) (0.58)	0.10 (-0.05, 0.24) (0.19)	0.17 (-0.03, 0.37) (0.09)	0.04 (-0.16, 0.23) (0.71)	0.13 (-0.04, 0.30) (0.12)	0.13 (-0.11, 0.37) (0.30)
IL-2	**-0.11 (-0.22**,** -0.00) (0.04)**	0.04 (-0.11, 0.20) (0.57)	-0.02 (-0.32, 0.29) (0.90)	0.03 (-0.21, 0.27) (0.79)	0.27 (-0.02, 0.55) (0.07)	0.03 (-0.28, 0.34) (0.85)	0.16 (-0.03, 0.35) (0.10)	0.20 (-0.04, 0.43) (0.10)
Anti-inflammatory								
IL-4	-0.09 (-0.20, 0.03) (0.15)	0.03 (-0.11, 0.17) (0.63)	-0.00 (-0.19, 0.19) (0.97)	0.15 (-0.01, 0.30) (0.06)	0.16 (-0.09, 0.41) (0.20)	**0.26 (0.05**,** 0.46) (0.01)**	0.13 (-0.04, 0.31) (0.13)	-0.12 (-0.38, 0.14) (0.35)
IL-10	0.03 (-0.10, 0.16) (0.63)	0.09 (-0.11, 0.29) (0.36)	0.03 (-0.14, 0.20) (0.74)	0.02 (-0.16, 0.21) (0.80)	0.05 (-0.19, 0.29) (0.68)	0.07 (-0.34, 0.48) (0.74)	0.14 (-0.05, 0.33) (0.14)	0.03 (-0.18, 0.23) (0.80)
IL-13	-0.02 (-0.11, 0.07) (0.68)	0.06 (-0.16, 0.28) (0.58)	-0.04 (-0.34, 0.26) (0.80)	**0.16 (-0.00**,** 0.33) (0.05)**	0.19 (-0.07, 0.44) (0.14)	0.09 (-0.09, 0.28) (0.32)	0.05 (-0.14, 0.23) (0.60)	0.02 (-0.24, 0.28) (0.88)
TGFb	**0.62 (0.04**,** 1.20) (0.04)**	0.06 (-0.69, 0.81) (0.87)	-0.12 (-1.06, 0.83) (0.80)	**0.58 (0.03**,** 1.13) (0.04)**	0.24 (-0.57, 1.06) (0.55)	0.42 (-0.51, 1.35) (0.37)	0.31 (-0.27, 0.89) (0.29)	-0.00 (-0.61, 0.61) (0.99)

*p* values are not adjusted for multiplicity. Observations lower than the detection limit were substituted by one-half of the corresponding lower detection limit; Boldface values indicate *p *<0.05

### Comparisons of the effects of APA on cytokines between groups

One-way ANOVA was used to assess between-group differences in cytokine changes at each time point. At T2, significant differences were observed for IFN-γ (F = 4.145, *p* = 0.0175), IL-6 (F = 4.261, *p* = 0.0156), and IL-8 (F = 4.295, *p* = 0.0151) (Table 3), with the T-APA group showing greater reductions in these pro-inflammatory cytokines compared with the NT-APA and control groups. This finding indicates a differential response to the interventions. However, at M1 and M3, no significant between-group differences were detected (*p* > 0.05), suggesting that the early differences in cytokine levels were not sustained over time.

**Table 3:** Between-group comparisons of the change from baseline,$\:F$value, and *p* value

Cytokine	Global test of no difference between groups at T2	Global test of no difference between groups at M1	Global test of no difference between groups at M3
IFNg	4.145 (0.0175)*	--	--
IL-12(p70)	--	--	0.01 (0.922)
IL-1a	0.577 (0.563)	0.133 (0.875)	--
IL-6	4.261 (0.0156)*	0.268 (0.776)	--
IL-8	4.295 (0.0151)*	--	--
IL-4	--	--	3.623 (0.061)
TGFb	0.332 (0.718)	--	--

Between-group comparisons were performed via ANOVA

*: *p* value < 0.05

### Correlations among cytokines and self-reported outcomes

Spearman’s rank correlation coefficient was used to examine the associations between changes in inflammatory biomarkers and changes in pain and function at T2, M1, and M3 (Table 4). In the T-APA group, IL-2 was positively correlated with pain at T2 (*p* = 0.30, *p* < 0.05) and M3 (*p* = 0.40, *p* < 0.05). Although improvements in pain and function were statistically significant, their magnitude should be interpreted within the context of established MCID (≥1.5 points for pain intensity reduction on NRS and ≥2.5 points for improvement on the RMDQ) [17]. IL-8 was negatively correlated with function (RMDQ change scores) at M1 (ρ = -0.38, *p* < 0.01), suggesting that greater reductions in IL-8 were associated with higher RMDQ scores (worse function). With the substantial attrition and missing data, this association should be interpreted cautiously. Previous studies on auricular therapies and cytokine modulation have shown inconsistent associations with IL-8 [11, 13], suggesting that this finding should be viewed as exploratory and hypothesis-generating rather than confirmatory.

**Table 4:** Correlations among changes in function and pain from baseline and changes in inflammatory biomarkers from baseline in all three groups at the T2, M1, and M3 follow-ups

Time		Changes from baseline	T-APA		NT-APA		CG	
			Function	Pain	Function	Pain	Function	Pain
T2	Pro-inflammatory	IL-1a	-0.09	0.12	-0.01	0.12	-0.02	-0.12
		IL-1b	0.06	0.08	0.08	0.03	-0.02	**-0.36** ^**b**^
		IL-2	0.09	**0.30** ^**c**^	0.12	0.04	-0.17	-0.22
		IL-6	0.15	0.01	-0.23	-0.09	0.07	**-0.36** ^**b**^
		IL-8	-0.16	0.20	-0.08	0.06	-0.06	**-0.28** ^**c**^
		IL-12(p40)	0.06	0.04	0.06	0.04	0.2	-0.13
		IL-12(p70)	-0.23	0.01	-0.06	-0.15	-0.04	-0.20
		TNFa	0	0.16	-0.04	0	-0.02	-0.17
		IFNg	-0.01	0.20	0.11	-0.08	0.07	-0.16
		IL-17 A	-0.20	0.07	-0.14	-0.07	0.04	-0.01
	Anti-inflammatory	IL-4	-0.05	0.02	-0.22	-0.15	0.06	-0.09
		IL-10	0.16	0.21	0.16	**0.30** ^**c**^	0.14	**-0.28** ^**c**^
		IL-13	0.07	0.06	-0.09	-0.05	-0.14	0.02
		TGFb	0.09	0.02	-0.07	-0.07	-0.06	-0.05
M1	Pro-inflammatory	IL-1a	0.04	0.08	0.02	-0.08	0.11	-0.10
		IL-1b	0.05	-0.04	0.13	-0.02	0.09	-0.22
		IL-2	0.12	0.17	0.05	0.23	-0.02	-0.08
		IL-6	0.01	0.24	-0.22	0.15	0.14	-0.03
		IL-8	**-0.38** ^**b**^	0.02	-0.28	0.14	-0.13	-0.07
		IL-12(p40)	-0.20	0	-0.16	0.04	0.13	-0.01
		IL-12(p70)	-0.03	0.01	0.21	0.06	-0.11	-0.04
		TNFa	0.23	**0.29** ^**c**^	-0.09	-0.01	0.13	0.08
		IFNg	0.15	0.12	0.09	-0.11	0.10	0.09
		IL-17 A	-0.01	0.10	0	-0.18	-0.09	-0.01
	Anti-inflammatory	IL-4	0.03	-0.07	-0.21	-0.05	0.08	0.21
		IL-10	0.11	-0.15	0.04	0.17	-0.02	-0.25
		IL-13	0.13	-0.02	0.05	0	0.08	0.07
		TGFb	0	0.04	0.01	-0.21	-0.01	0.03
M3	Pro-inflammatory	IL-1a	-0.14	0.16	-0.06	0.20		
		IL-1b	0.02	0.07	-0.18	-0.02		
		IL-2	0.09	**0.40** ^**c**^	-0.10	0.22		
		IL-6	-0.05	0.11	-0.06	**0.44** ^**b**^		
		IL-8	0.12	0.21	-0.04	**0.47** ^**b**^		
		IL-12(p40)	0.21	0.11	-0.04	0.16		
		IL-12(p70)	0.13	0.22	0.18	**0.36** ^**c**^		
		TNFa	0.06	0.09	0.05	0.23		
		IFNg	-0.07	-0.05	-0.13	-0.01		
		IL-17 A	-0.19	0.07	-0.32	0.19		
	Anti-inflammatory	IL-4	-0.07	0.15	0.21	0.33		
		IL-10	0.25	-0.01	0.11	0.09		
		IL-13	-0.07	0.03	0	-0.04		
		TGFb	-0.05	-0.01	0.13	0.14		

Spearman’s rank ^a^*p *value < 0.0001; ^b^*p *value < 0.01; ^c^*p* value < 0.05; Boldface numbers indicate statistically significant results

The NT-APA group demonstrated a delayed proinflammatory response, with the levels of IL-6 (*p* = 0.44, *p* < 0.01), IL-8 (*p* = 0.47, *p* < 0.01), and IL-12 (p70) (ρ = 0.36, *p* < 0.05) at M3 being positively correlated with pain. Additionally, IL-10 was positively correlated with pain at T2 (ρ = 0.30, *p* < 0.05), suggesting potential involvement in immune modulation. The control group showed minimal significant correlations, suggesting that inflammation-related changes in pain and function were less pronounced without intervention. (Table 4).

### Differentially expressed gene analysis

#### RNA sequencing (RNA-seq) exploration

In the comparison of M1 vs. T1 in the T-APA group, a total of 302 genes presented an absolute fold change ≥ 1.5, and *CXCL8* was significantly upregulated, with an absolute fold change of 1.8965 and an adjusted p value of 0.0312. When M1 was compared with T2 in the T-APA group, *GPS2* was significantly downregulated, with an absolute fold change of 0.3888 and an adjusted p value of 0.0178. Although *RSAD2* was upregulated with an absolute fold change of 1.5812 and an adjusted p value of 0.0723, it was not statistically significant (Table 5). Moreover, no genes were found to be significantly differentially expressed between T2 and T1 within the T-APA group or between the T-APA and control groups at the T1, T2, and M1 time points. These findings provide insights into the molecular changes associated with APA treatment in cLBP patients and highlight specific gene expression alterations that may correlate with observed clinical outcomes. 

**Table 5:** Significantly differentially expressed genes in the T-APA group

Gene	log2FoldChange	Fold Change	Direction	Adjusted *p* value
*CXCL8*	0.9234	1.8965	upregulated (M1 vs. T1)	0.0312
*GPS2*	-1.3630	0.3888	downregulated (M1 vs. T2)	0.0178
*RSAD2*	0.6610	1.5812	upregulated (M1 vs. T2)	0.0723

#### Quantitative PCR validation

**Fig. 2:**
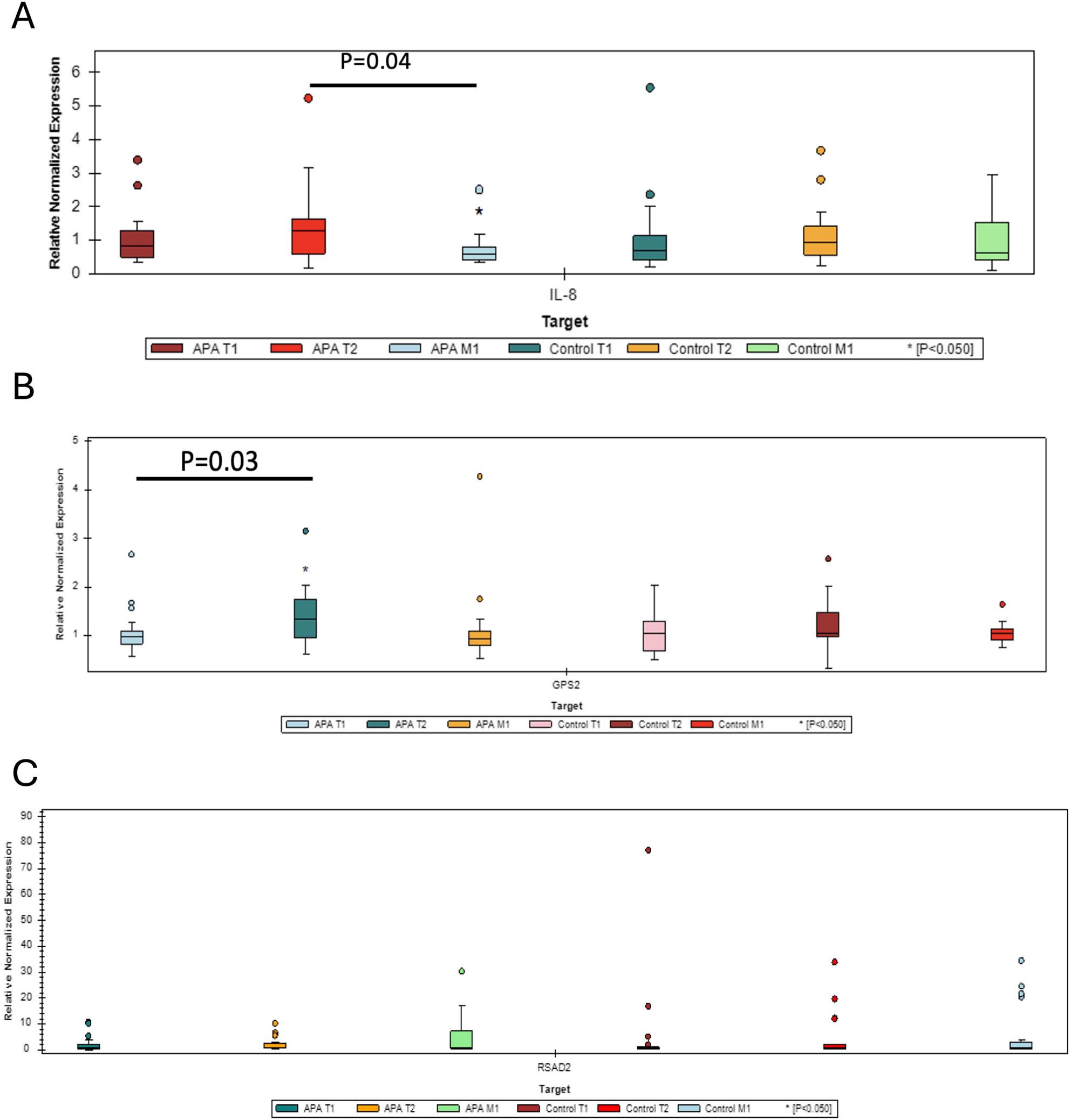
Relative normalized expression of IL-8 across different time points and groups. CXCL8 expression was significantly lower at M1 than at T2 in the T-APA group (**A**), and GPS2 expression was significantly greater at T2 than at T1 at baseline (**B**) in the T-APA group. No significant changes in RSAD2 expression were observed at any time point within the T-APA or control groups or between the APA and control groups (**C**)

The qPCR results revealed downregulation of *CXCL8* in the M1 vs. T2 comparison (fold change = 0.60, p value = 0.042) and upregulation of GPS2 (fold change = 1.30, p value = 0.033) in the T2 vs. T1 comparison (Fig. 2). No significant changes in the expression of IL-8 or *GPS2* were observed at other time points within the T-APA or control groups or between the T-APA and control groups at all three time points. No significant changes in *RSAD2* expression were observed at any time point within the T-APA or control groups or between the APA and control groups. These qPCR results confirmed that the changes in the expression of a group of genes were associated with APA treatment in cLBP patients.

## Discussion

This study provides supporting evidence that APA modulates inflammatory cytokines in older adults suffering from cLBP. The T-APA intervention led to notable improvements in pain intensity and physical function, which were correlated with a decrease in proinflammatory cytokines, including IL-2, IL-8, and TNF-α, and an increase in the anti-inflammatory cytokine TGF-β. These findings suggest that the therapeutic effects of APA are likely mediated through its influence on the immune system, specifically by altering the balance of pro- and anti-inflammatory cytokines. In contrast, the NT-APA group, which served as an active control intervention, did not show similar improvements or proinflammatory cytokine changes, suggests the specificity of the targeted approach. While these results provide preliminary support for the immunomodulatory effects of APA, they should be interpreted cautiously given the exploratory design and multiple cytokine comparisons, which increase the potential for Type I error. Nevertheless, these results contribute to the growing body of evidence supporting APA as a feasible, noninvasive, and low-cost complementary intervention for managing chronic pain, particularly among older adults who may have limited tolerance for conventional pharmacological treatments.

As this manuscript is a secondary analysis focused on cytokine and gene expression mechanisms, the MCID thresholds were referenced to provide interpretive context rather than applied as analytic cutoffs. Although participants demonstrated significant reductions in pain intensity and improvements in physical function, these findings were not evaluated against MCID benchmarks established in the parent randomized controlled trial by Kawi et al. (2025), where a ≥ 1.5-point reduction in pain and a ≥ 2.5-point improvement in function were defined as clinically meaningful. We instead emphasize the biological mechanisms underlying APA’s effects, exploring how cytokine modulation may contribute to the observed symptom improvements. Future studies with larger and more diverse samples are needed to further examine these mechanistic relationships and confirm whether they correspond with sustained, clinically meaningful outcomes.

At one month (T2) after treatment, we observed reduced IL-8 protein levels. Similarly, the expression of the *CXCL8* gene, which encodes the IL-8 protein, also decreased by the second month (M1) compared with its level at one month (T2). This sequential reduction suggests that the treatment may initially lower IL-8 protein levels, followed by a subsequent downregulation of *CXCL8* gene expression. The regulation of *CXCL8/IL-8* gene expression indicates that post-transcriptional modifications and feedback mechanisms play significant roles in controlling cytokine levels [25]. The delayed decrease in gene expression found in our study could reflect a feedback mechanism or the time required for the treatment to impact transcriptional regulation. This sequence of events is important because it implies that the treatment not only has an immediate effect on reducing inflammation but also may lead to sustained suppression of the inflammatory pathway over time.

Moreover, we found that *GPS2* levels increased one month after treatment, which coincided with the downregulation of proinflammatory cytokines. *GPS2* is crucial for regulating inflammation, particularly by acting as a negative regulator in the cytoplasm. It inhibits the proinflammatory TNF-alpha pathway, helping to modulate inflammatory responses [26]. The observed increase in *GPS2* might reflect the body’s adaptive response to APA treatment, which aims to restore balance in the inflammatory environment. As *GPS2* levels increase, its inhibitory effect on the TNF-alpha pathway could contribute to the observed reduction in proinflammatory cytokines. These findings suggest that *GPS2* could be part of a feedback mechanism that suppresses excessive inflammation following treatment, highlighting its potential role in maintaining immune homeostasis.

The discrepancies in gene expression for CXCL8 and GPS2 between RNA-seq and qPCR likely stem from technical and biological factors, as well as differences in time-point comparisons. RNA-seq revealed that IL-8 (CXCL8) was upregulated at M1 compared with T1 at baseline, whereas qPCR revealed a reduction at M1 compared with T2. Similarly, RNA-seq revealed GPS2 downregulation at M1 compared with T2, whereas qPCR revealed upregulation at T2 compared with T1 at baseline. These variations reflect the dynamic nature of gene expression over time and differences in the specific comparisons being analyzed. Additionally, technical differences between RNA-seq and qPCR, such as sensitivity, primer design, data normalization, and biological factors such as mRNA stability and feedback regulation, further contribute to these results. Together, these factors highlight the need for complementary methods to validate and interpret gene expression data.

For gene expression, we observed discrepancies between the RNA-seq and qPCR results, with opposite results for both *CXCL8* and *GPS2*. One potential reason is the relatively small sample size used for RNA-seq, which limits statistical power and increases the likelihood of false positives, particularly when low-fold changes in gene expression are detected [27]. RNA-seq often identifies subtle changes in expression that may not be robust enough to validate with qPCR, a method with higher sensitivity for specific targets but less suited to detecting subtle genome-wide variations. Furthermore, alternative splicing may also play a role in these discrepancies. RNA-seq captures all transcript isoforms of a gene, which can lead to an aggregate expression signal that includes spliced variants. In contrast, qPCR typically targets a specific transcript, which might explain the differences in the observed expression levels. These discrepancies highlight the importance of understanding the technical and biological contexts when comparing gene expression data from different platforms.

Although the RNA-seq analysis was limited by a small sample size (*n* = 9), it was intentionally designed as an exploratory, hypothesis-generating step to identify potential molecular targets that mirror the cytokine and protein-level changes observed in the larger cohort. Participants were selected based on stringent inclusion criteria, which is those with complete data across all three time points to ensure biological consistency and minimize variability. Importantly, the RNA-seq findings were validated in an independent, larger cohort (*n* = 40) using quantitative PCR, confirming significant expression changes in key genes (*CXCL8* and *GPS2*) that were consistent with the directionality observed in the RNA-seq data. This two-step approach, including initial exploration and validation, supports the biological relevance and reproducibility of the identified molecular signatures despite the limited RNA-seq sample size.

The findings from this study align with and expand upon previous research examining the efficacy of auricular therapies in pain management. Studies have demonstrated that auricular acupuncture and acupressure can significantly reduce pain and inflammation, suggesting that these therapies exert their effects by modulating the immune response [9, 11, 13]. Our study adds to this body of work by providing direct evidence of cytokine modulation, thereby offering a potential mechanistic explanation for the observed clinical benefits. Compared with other studies, our research focused on an older population with cLBP, a demographic that is often underrepresented in pain management studies.

Studies using functional MRI to explore the neural mechanisms of auricular acupuncture have identified specific brain regions activated by auricular stimulation that are associated with pain relief [28]. Although our study did not include neuroimaging, the observed changes in cytokine levels suggest that similar neural-immune pathways might be involved in the efficacy of APA. This finding reinforces the hypothesis that the ear microsystem can influence systemic physiological functions by connecting with specific brain areas.

Despite these promising results, our study has several limitations that should be acknowledged. First, the relatively short follow-up period (up to 3 months) may not fully capture the long-term effects of APA on cytokine levels and pain outcomes. Second, as a mechanistic secondary analysis, we did not reapply MCID criteria in the statistical models, since we focused on exploring biological mechanisms rather than reassessing clinical response thresholds. Third, the high attrition rate and missing biomarker data may have introduced bias and reduced the robustness of cytokine analyses, especially regarding IL-8 and its association with functional improvement. Fourth, the concurrent use of analgesic medications, including opioids, may have influenced both cytokine responses and perceived symptom relief; however, these effects could not be analyzed separately due to variability in dosage, duration, and medication type among participants. In addition, the RNA-seq analysis was conducted in a small exploratory subset (*n* = 9; six in the T-APA and three in the control group), which limits statistical power and generalizability.

Analytical limitations should also be considered when interpreting these findings. The exploratory nature of the analysis and the presence of missing cytokine data necessitated a nonparametric two-step approach rather than a mixed-effects model, limiting the estimation of group-by-time interaction effects. Sensitivity analyses were conducted with and without missing data imputation to assess the potential influence of incomplete data; however, outlier exclusion was not performed to avoid further reducing power given the small sample size. Finally, because p-values were not adjusted for multiple comparisons, the possibility of Type I error cannot be excluded. These analytical constraints, combined with the limited sample size, warrant cautious interpretation of the results until validated in larger, confirmatory studies.

Future studies should aim to include larger, more diverse samples and extend the follow-up period to better understand the durability of the effects of APA. Furthermore, analyses should control for or stratify by concurrent medication use, particularly opioids, to clarify the independent biological and clinical effects of APA. While our study focused on cytokine changes as a marker of APA efficacy, other biological pathways, such as neuropeptide modulation or gene expression changes, might also play significant roles. Studies suggested that neuropeptides such as β-endorphin may mediate the analgesic effects of auricular therapies [15, 29]. Integrating these aspects into future research could provide a more comprehensive understanding of the mechanisms underlying APA.

In conclusion, this study contributes valuable insights into the biological mechanisms of APA, reinforcing its potential as a viable treatment for cLBP. The observed modulation of inflammatory cytokines provides a plausible explanation for the clinical benefits of APA. It underscores the importance of mechanistic research in advancing the integration of complementary therapies into mainstream healthcare. As the evidence base for APA and similar interventions continues to grow, their acceptance and utilization will increase in clinical settings, offering patients more options for managing chronic pain. Although the observed improvements in pain and function are promising, future studies with larger samples should confirm whether these changes reach clinically meaningful levels.

## Supplementary Information

Supplementary Material 1.

## Data Availability

The datasets generated and analyzed during the current study are available from the corresponding author upon reasonable request. Restrictions may apply due to institutional policies and ethical considerations regarding participant privacy.

## Abbreviations

APA: Auricular Point Acupressure
cLBP: Chronic Low Back Pain
RCT: Randomized Controlled Trial
T: APA–Targeted Auricular Point Acupressure
NT: APA–Non–Targeted Auricular Point Acupressure
CG: Control Group
IL: Interleukin (e.g., IL–2, IL–6, IL–8)
TNF: α–Tumor Necrosis Factor–alpha
TGF: β–Transforming Growth Factor–beta
CXCL8: C–X–C Motif Chemokine Ligand 8 (also known as IL–8)
GPS2: G Protein Pathway Suppressor 2
RNA: seq–RNA Sequencing
qPCR: Quantitative Polymerase Chain Reaction
NRS: Numerical Rating Scale
RMDQ: Roland–Morris Disability Questionnaire
EDTA: Ethylenediamine Tetraacetic Acid
QC: Quality Control
GRCh38.83: Human Genome Reference Consortium Build 38, Version 83
HISAT2: Hierarchical Indexing for Spliced Alignment of Transcripts 2
DESeq2: Differential Expression Analysis Package in R
GLM: Generalized Linear Model
ANOVA: Analysis of Variance
M1: One–month Follow–up
M3: Three–month Follow–up
IFN: γ–Interferon–gamma
ACTB: Beta–actin (Housekeeping Gene)
SMD: Standardized Mean Difference
IRB: Institutional Review Board
PAXgene: PreAnalytix Blood Collection and RNA Stabilization System
SPSS: Statistical Package for the Social Sciences
CI: Confidence Interval
IQR: Interquartile Range
