# Supplementary figures and images for: Effects of auricular point acupressure on inflammatory signaling in chronic low back pain: a secondary analysis of a randomized controlled trial
Source: BMC Complement Med Ther. 2025 Dec 29;26:35. doi: 10.1186/s12906-025-05227-9 (PMC12860021; doi:10.1186/s12906-025-05227-9)

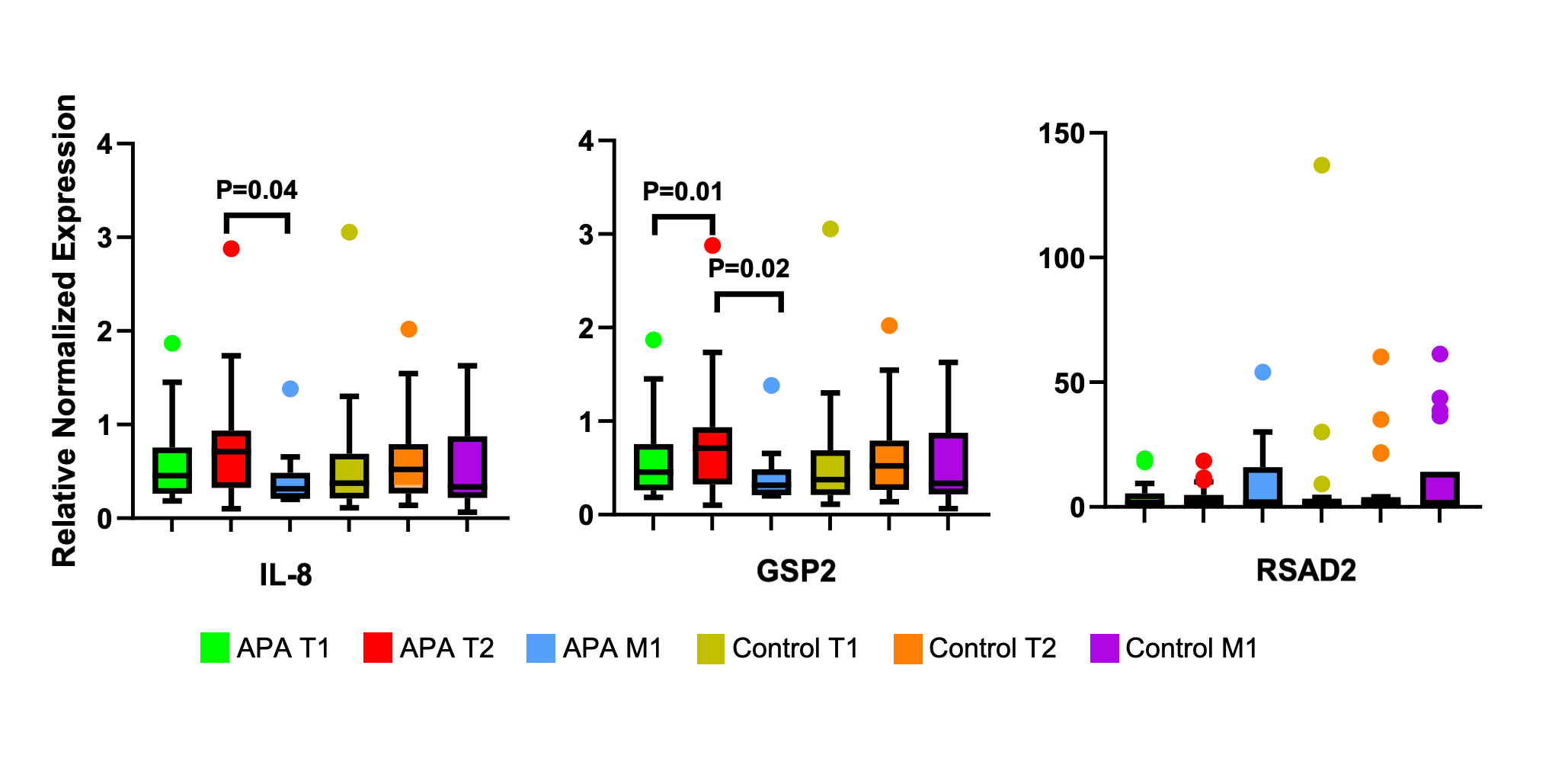

Supplement: Supplementary file 1 — Supplementary Material 1. [file 12906_2025_5227_MOESM1_ESM.png]
